# Bacterial community in naturally fermented milk products of Arunachal Pradesh and Sikkim of India analysed by high-throughput amplicon sequencing

**DOI:** 10.1038/s41598-018-19524-6

**Published:** 2018-01-24

**Authors:** H. Nakibapher Jones Shangpliang, Ranjita Rai, Santosh Keisam, Kumaraswamy Jeyaram, Jyoti Prakash Tamang

**Affiliations:** 1 DAILAB (DBT-AIST International Laboratory for Advanced Biomedicine), Bioinformatics Centre, Department of Microbiology, School of Life Sciences, Sikkim University, Gangtok, 737102 India; 2 Microbial Resources Division, Institute of Bioresources and Sustainable Development (IBSD), Takyelpat Institutional Area, Imphal, 795 001 Manipur India

## Abstract

Naturally fermented milk (NFM) products are popular ethnic fermented foods in Arunachal Pradesh and Sikkim states of India. The present study is the first to have documented the bacterial community in 54 samples of NFM products viz. *chhurpi*, *churkam, dahi* and *gheu*/*mar* by high-throughput Illumina amplicon sequencing. Metagenomic investigation showed that *Firmicutes* (*Streptococcaceae*, *Lactobacillaceae*) and *Proteobacteria* (*Acetobacteraceae*) were the two predominant members of the bacterial communities in these products. *Lactococcus lactis* and *Lactobacillus helveticus* were the predominant lactic acid bacteria while *Acetobacter* spp. and *Gluconobacter* spp. were the predominant acetic acid bacteria present in these products.

## Introduction

Naturally fermented milk (NFM) products are prepared by one of the oldest processes of milk fermentation in the world using raw or boiled milk to ferment spontaneously or by back-sloping method^1^. Some naturally fermented milk products are *chhu, chhurpi, dahi, lassi, misti dahi, mohi, philu, shoyu, somar* and *srikhand* (cow/buffalo/yak milk) of India, Nepal, Pakistan, Bhutan and Bangladesh^2–5^, *kurut* of China^6^, *aaruul, airag, byasulag, chigee*, *eezgii, khoormog* and *tarag* of Mongolia^7–9^, *ergo* of Ethiopia, *kad, lben, laban, rayeb, zabady, zeer* of Morocco and Northern African and Middle East countries, *rob* (from camel milk), *biruni* (cow/camel milk), *mish* (cow/camel milk) of Sudan, *amasi* (*hodzeko, mukaka wakakora*) of Zimbabwe, *nunu* (from raw cow milk) of Ghana and *kule naoto* of Kenya^10,11^, *filmjölk* and *långfil* of Sweden^12^, *koumiss* or *kumis* or *kumys* or *kymys* of the Caucasian area^13^. Various cultivation-based studies reported lactic acid bacteria as the predominant microbiota present in the NFM products of the world mostly *Lactococcus lactis* subsp. *cremoris, Lc. lactis* subsp. *lactis*, *Lactobacillus casei/Lb. paracasei, Lb. fermentum, Lb. helveticus*, *Lb. plantarum, Lb. acidophilus, Lb. coryniformis, Lb. curvatus*, *Lb. kefiranofaciens, Lb. kefiri, Lb. buchneri*, *Lb. jensenii, Lb. kitasatonis, Enterococcus faecium, E. faecalis* and *Leuconostoc mesenteroides*, *Streptococcus thermophilus*, and others^11,14–19^. Besides bacteria, yeasts are also present in some NFM products which include *Candida lusitaniae, C. parapsilosis, C. rugosa, C. tropicalis, Kluyveromyces marxianus, Saccharomyces cerevisiae, Galactomyces geotrichum, Issatchenkia orientalis, Kazachstania unispora, Pichia mandshurica*, *P. fermentans, P. kudriavzevii*, and others^8,11,13,16,20–22^.

**Figure 1:**
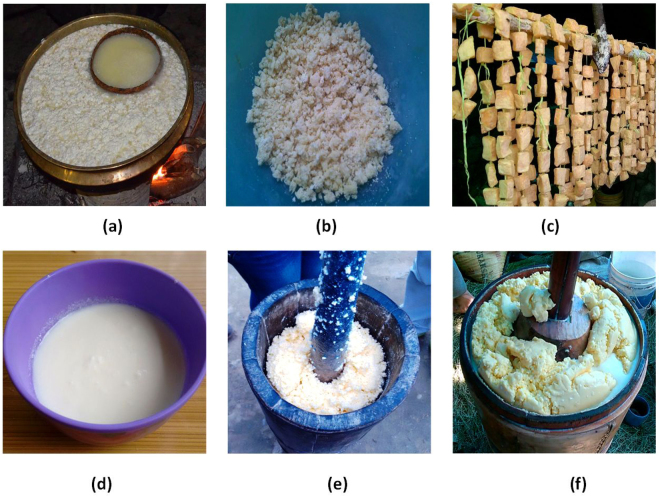
(**a**) *Chhurpi* of Arunachal Pradesh (AP); (**b**) *Chhurpi* of Sikkim; (**c**) *Churkam* of AP; (**d**) *Dahi* of Sikkim; (**e**) *Gheu* of Sikkim; (**f**) *Mar* of AP.

High altitude (upto 4878 m)-naturally fermented milk products of cow (*Bos taurus*) or yak (*Bos grunniens*)-milk prepared by back-sloping are common in the Himalayan states of Arunachal Pradesh and Sikkim in India which include *chhurpi*, *churkam, dahi* and *gheu*/*mar* (Fig. 1a–f) as a protein-rich food supplement and also as a source of livelihood^5^. *Dahi*, similar to yogurt, is the first product of milk fermentation by back-sloping, and is consumed as savory non-alcoholic beverage. *Gheu*/*mar* (crude butter) is a fat-rich milk product obtained by a process of milk churning in which the casein-rich soft-variety product called *chhurpi* (cottage cheese-like) is produced, and is consumed as curry/soup in meals; and c*hurkam* (hard-variety of *chhurpi*) is the product of dehydrated *chhurpi*, which is used as masticatory as chewing gum in high altitudes. Lactic acid bacteria were predominant with the load of 10^8^ cfu/g in the Himalayan fermented milk products^17^. *Lactobacillus bifermentans, Lb. alimentarius, Lb. paracasei* subsp. *pseudoplantarum, Lactococcus (Lc.) lactis* subsp. *lactis, Lc. lactis* subsp. *cremoris*; *Lb. plantarum, Lb. curvatus, Lb. fermentum, Lb. kefir, Lb. hilgardii, Enterococcus faecium* and *Leuconostoc mesenteroides* were reported from *dahi* and *chhurpi* of Sikkim based on phenotypic, biochemical characterization and mol (%) content of G+C of DNA^14,17^. However, no study has been conducted yet on *churkam* and *gheu*/*mar*.

As it is well known that the cultivability of microbiota is still a limiting factor in understanding the natural food fermentation^23,24^, application of high throughput metagenomic techniques like Illumina amplicon sequencing may serve to give more insight into microbial ecology of natural food fermentation. Metagenomic studies of various fermented milk products like kefir, buttermilk, cheeses etc have shown a realistic view of the microbial community structure involved in the natural milk fermentation^21,24–28^. In this study we aimed to anlayse the bacterial community structure of fifty-four samples of naturally fermented milk products (*chhurpi, churkam, dahi* and *gheu*/*mar*) of Arunachal Pradesh and Sikkim by Illumina amplicon sequencing. This is the first report on bacterial community in NFM products of the Himalayas using in-depth metagenomic analysis.

## Results

### Overall microbial community structure

**Figure 2:**
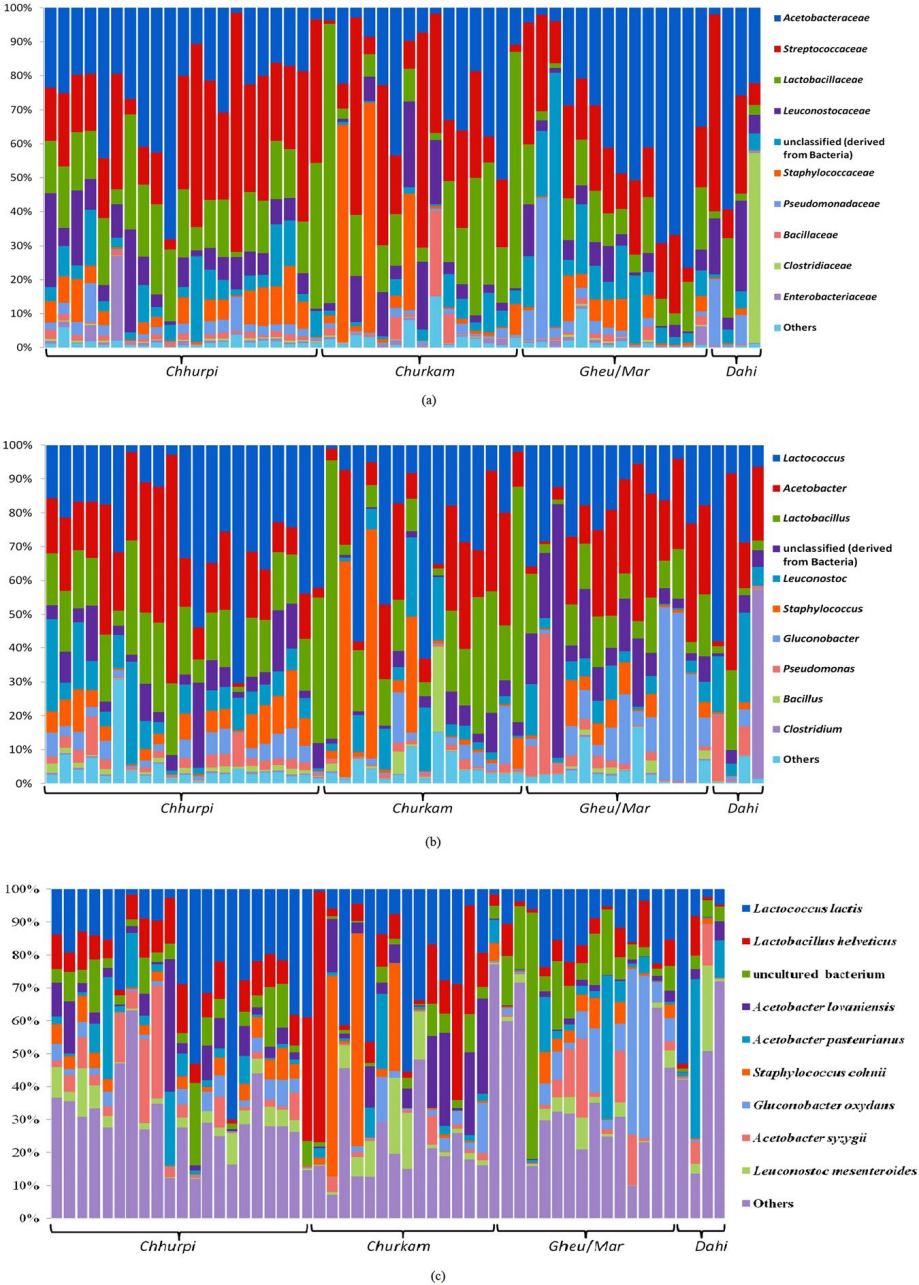
The overall bacterial composition of NFMs: *chhurpi*, *churkam*, *gheu*/*mar* and *dahi* at different taxonomic levels (**a**) Family, (**b**) Genus and (**c**) Species.

The bacterial composition of the different naturally fermented milk products (*chhurpi, churkam, dahi* and *gheu*/*mar*) was compared at different taxonomic levels (Fig. 2a–c). The bacterial phyla present in four types of NFM products were *Firmicutes* and *Proteobacteria*, respectively (data not shown). Phylum *Firmicutes* was represented by six families belonging to *Streptococcaceae* (24.2%), *Lactobacillaceae* (16.8%), *Leuconostocaceae* (8.0%), *Staphylococcaceae* (6.8%), *Bacillaceae* (1.6%), and *Clostridiaceae* (1.3%); and phylum *Proteobacteria* included *Acetobacteraceae* (26.8%), *Pseudomonadaceae* (3.3%) and *Enterobacteriaceae* (1.2%) (Fig. 1a). The overall bacterial diversity of these NFM products were predominated by species belonging to the lactic acid bacteria: *Lactococcus lactis* (19.7%) and *Lactobacillus helveticus* (9.6%) and *Leuconostoc mesenteroides* (4.5%) (Fig. 2b,c). Additionally, species belonging to the acetic acid bacteria: *Acetobacter lovaniensis* (5.8%), *Acetobacter pasteurianus* (5.7%), *Gluconobacter oxydans* (5.3%), and *Acetobacter syzygii* (4.8%) were also observed (Fig. 2b,c). The percentage of *Enterobacteriaceae* was 1.2% (Fig. 2a), whereas the percentage of genus *Enterococcus* was below 0.5% (data not shown), hence it was not shown at the genus level (Fig. 2b). Percentage of *Streptococcus thermophilus* was below 0.1% (data not shown). The percentage of unclassified bacteria at the taxonomical levels was 7.9% (Fig. 2a–c). Presence of uncultured bacterium was shown in all samples (Fig. 2c).

### Multivariate analysis

**Figure 3:**
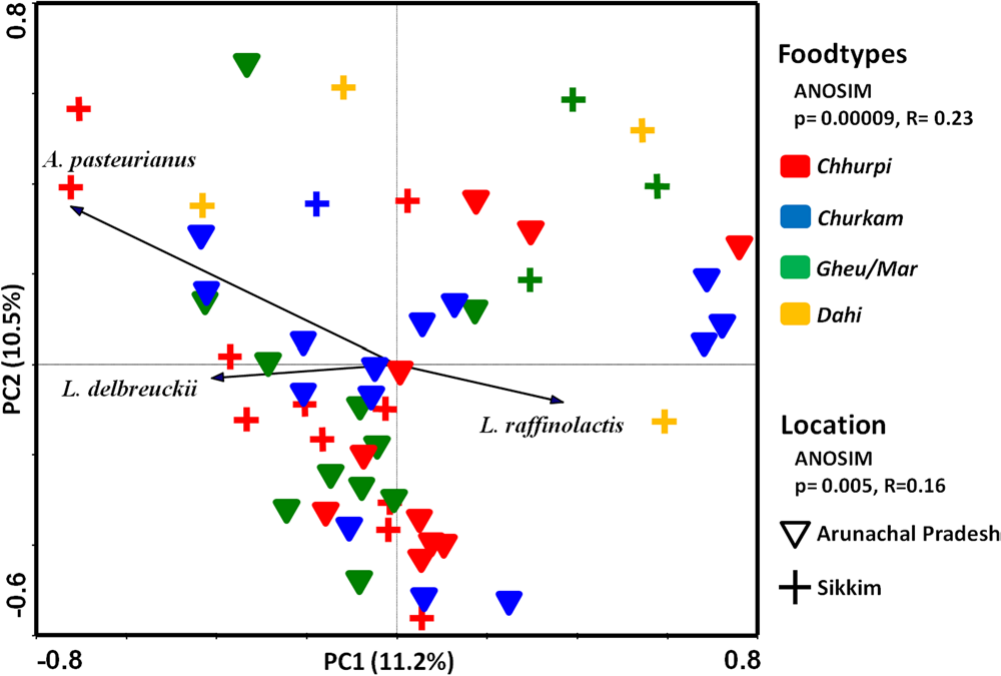
PCA plot shows the difference in bacterial community structure among the NFM products of Arunachal Pradesh and Sikkim. Arrow indicates the species direction. Significant difference is shown by ANOSIM analyzed with 10,000 permutations using Bray-Curtis distances.

PCA using species-level OTUs data showed significant differences among the NFM products studied (Fig. 3). The NFM products collected from two regions (Arunachal Pradesh and Sikkim) showed significant difference in the bacterial community structure (ANOSIM, p = 0.005, R = 0.16), but however, there was no significant difference between the same products prepared from different sources of milk (cow or yak). This reflects the regional contribution to the bacterial diversity of these products with respect to their location of preparation, but not from the milk source whereby these products are being prepared.

### Alpha diversities

**Figure 4:**
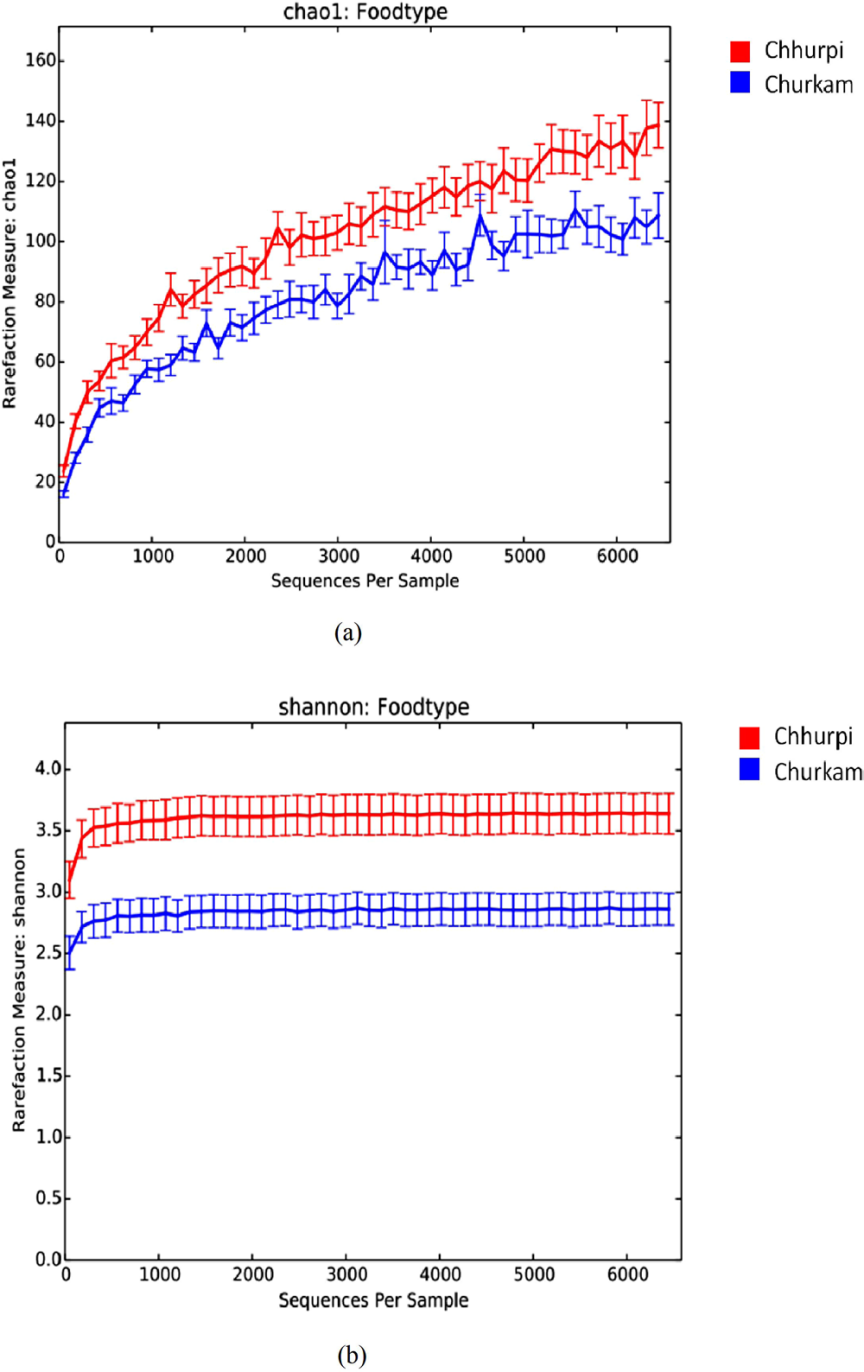
Difference in the bacterial alpha diversity indices of *chhurpi* and *churkam* (**a**) Chao1 species richness and (**b**) Shannon Diversity Index.

**Figure 5:**
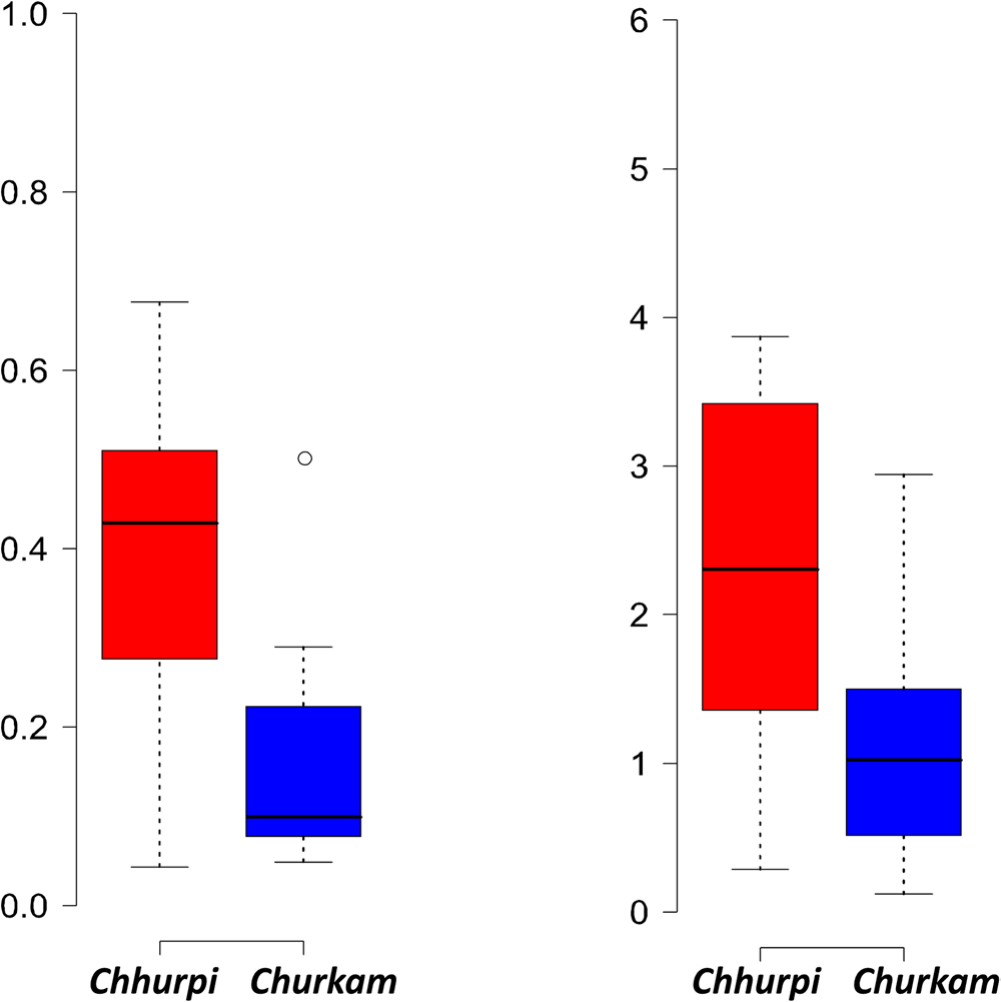
Boxplot showing the difference in the relative abundance of (**a**) *Clostridiaceae* and (**b**) *Pseudomonadaceae* between *chhurpi* and *churkam*.

Alpha diversities were compared on the basis of states (Sikkim and Arunachal Pradesh)/places of collection of samples, animal’s milk source (cow/yak) and product types (Table 1). There was no significant difference between the states/regions and animal’s milk source, respectively. However, significance difference (p = 0.0125) was observed in terms of product types i.e., *chhurpi* and *churkam* in Chao1 species richness (Fig. 4). *Chhurpi* and *churkam* are two final products of milk fermentation where the latter is produced through a process of dehydration of the former and is usually kept for a longer fermentation. Multivariate analysis of species level OTUs showed a significant difference (ANOSIM p = 0.002, R = 0.16) between the two products. However, there is no significant difference among the general fermenting bacteria. Also, we observed a significant difference in *Clostridiaceae* (p = 0.0004) and *Pseudomonadaceae* (p = 0.013) between these two food types (Fig. 5).

**Table 1:** Alpha diversity profiles of NFM products of India.

Group1	Group 2	Group 1 mean	Group 1 std	Group 2 mean	Group 2 std	t stat	p-value
Chao1							
*Chhurpi*	*Dahi*	138.6654794	33.93332555	90.56944444	28.79901552	2.549699487	0.0152
*Chhurpi*	*Churkam*	138.6654794	33.93332555	108.6683546	26.9353883	2.695182315	0.0125
*Dahi*	*Gheu*	90.56944444	28.79901552	127.6180229	33.10848324	−1.91332029	0.0738
*Chhurpi*	*Gheu*	138.6654794	33.93332555	127.6180229	33.10848324	0.925146304	0.3583
*Churkam*	*Gheu*	108.6683546	26.9353883	127.6180229	33.10848324	−1.60079762	0.1171
*Dahi*	*Churkam*	90.56944444	28.79901552	108.6683546	26.9353883	−1.10004359	0.2864
**Shannon**							
*Chhurpi*	*Dahi*	3.639041175	0.736572535	2.657764997	0.378296426	2.493760723	0.0158
*Chhurpi*	*Churkam*	3.639041175	0.736572535	2.860086707	0.47435654	3.400743965	0.0022
*Dahi*	*Gheu*	2.657764997	0.378296426	3.339920996	0.823489314	−1.51693208	0.1459
*Chhurpi*	*Gheu*	3.639041175	0.736572535	3.339920996	0.823489314	1.089687949	0.2738
*Churkam*	*Gheu*	2.860086707	0.47435654	3.339920996	0.823489314	−1.82046908	0.0789
*Dahi*	*Churkam*	2.657764997	0.378296426	2.860086707	0.47435654	−0.73983568	0.4743

## Discussion

In this study, bacterial diversity was explored by barcoded Illumina MiSeq amplicon sequencing of the 16 S rRNA gene (V4-V5 region). The applied method using high throughput sequencing detected *Lactococcus lactis, Lb. helveticus, Acetobacter lovaniensis, A. pasteurianus, A. syzygii, Gluconobacter oxydans* and *Leuconoctoc mesenteroides* (above 1%) in all 4 samples of NFM products. Reads of OTUs in present study could not detect *Lb*. *farciminis*, *Lb*. *biofermentans*, *Lb*. *hilgardi*, *Lb*. *paracasei* subsp. *pseudoplantarum*, *Lb*. *hilgardii*, *Lb*. *paracasei* subsp. *paracasei* which were reported earlier in *chhurpi* and *dahi* based on limited phenotypic characterization^14,17^. However, *Lb. helveticus* (9.6%) was detected in the present culture-independent method which was not reported in culture dependent method earlier. *Lb. helveticus* is known to be present in dairy products^29^. A major composition of *Lactococcus lactis* (*Streptococcaceae*) and *Lb. helveticus (Lactobacillaceae)* was found to be the most predominant species along with *Leuc. mesenteroides* (*Leuconostocaceae*) in the NFM products of India, which still form what are commonly known as the primary cultures in milk fermentation^1^. Metagenomics-based studies of other milk products around the world like kefir, cheeses, have also reported to harbour species of *Lactobacillus*, *Lactococcus* and *Leuconostoc*^25,26,30,31^ as the dominant bacteria in general. Apart from the common known lactic acid bacteria group, a relatively high abundance of *Proteobacteria*-associated *Acetobacteraceae* (acetic acid bacteria) was observed in *gheu*/*mar* products. *Acetobacteraceae* members have also been reported in milk-related products^19,25,32,33^, and their dominance in *gheu/mar* (churned before heating) products than the subsequent downstream products (*chhurpi* and *churkam*) may be due to the effect of heating during the processing steps. Even though the *Acetobacteraceae* members were still present in *chhurpi* and *churkam*, the abundance was generally low. During the fermentation of *chhurpi* and *churkam*, we observed an increase in the abundance of *Streptococcaceae* (*Lactococcus*) and subsequently a build-up in the *Lactobacillaceae* (*Lactobacillus*) population in *churkam*.

Based on OTUs system, the percentage of *Enterobacteriaceae* and genus *Enterococcus* was very low in NFM samples analyzed. *Enterococcus faecalis, Ent. faecium* along with *Lactococcus lactis* subsp*. lactis* were reported from *dahi* of Bhutan based on 16 S rRNA gene sequencing^6^. *Nunu*, African NFM product, is frequently contaminated with pathogenic *Enterobacteriaceae*, demonstrated by short-read-alignment-based bioinformatics tools which may be used for high-throughput food safety testing^34^. *Staphylococcaceae*, *Bacillaceae*, *Clostridiaceae* and *Pseudomonadaceae* were observed at relatively low level in this study probably as contaminants. *Pseudomonadaceae* (*Pseudomonas fluorescens)* is usually present in milk and milk products as sources of contaminants^35^ and *Clostridiaceae* (*Clostridium tyrobutyricum*) is another bacterium found in cheese causing late blowing defect^36^. These contaminants were probably associated with the overall handling process, since samples are naturally fermented milk products, and there is no controlled process involved. Contamination of unwanted or rather non-fermenting bacteria are known to have acquired from various sources of production environment^37,38^. Presence of uncultured bacterium was shown in all samples analyzed. Uncultured bacterium group at species level were obtained using OTUs method, as the database could not assign them to any of their closest taxa. OTUs system put sequences into bins based on similarity of sequences within a data set to each other^39^. Moreover, limitations to using OTUs-based method is that the clustering algorithms are computationally intensive, relatively slow, and require significant amounts of memory^40^.

However, the predominance of few species were observed in a particular product showing the remarkable diversity of microbiota among 4 analyzed samples of NFM products and subsequently a build-up in the Lactobacillaceae (*Lactobacillus*) population in *churkam*. *Lactococcus lactis* was predominant in *chhurpi*, *dahi* and *churkam*, whereas in *gheu/mar* samples, it was relatively less. *Lb. helveticus* was dominant in *churkam* comparable to other 3 NFM products. However, *Leuc. mesenteroides* was predominant in *dahi* samples. Though we observed a fairly equal distribution between *Lactococcus* and *Acetobacter* species in 4 NFM products, however, at species level *Lactococcus* was represented only by *Lc. lactis* whereas *Acetobacter* was represented by *A. lovaniensis*, *A. pasteurianus*, *A. syzygii* and *Gluconobacter oxydans*. Diversity in bacterial species among the 4 NFM products was observed based on alpha diversity analysis. However, significance difference was observed only in between *chhurpi* and *dahi* (p = 0.0152) and *chhurpi* and *churkam* (p = 0.0125), respectively.

## Conclusion

Earlier reports on *chhurpi* and *dahi* of North East India was based on limited culture-dependent analysis with some species of lactic acid bacteria. However, in the present study the NGS data of *chhurpi*, *churkam, dahi* and *gheu* showed the abundance of *Lactococcus lactis* (*Streptococcaceae*), *Lb. helveticus (Lactobacillaceae)* with *Leuc. mesenteroides* (*Leuconostocaceae*) as one the main bacterial species which may be the reliable information on microbial profile of NFM products. The application of NGS culture-independent methods to study the microbial ecology of fermented foods is of great significance in understanding the products, where Illumina sequencing has been shown to be one of the reliable tools in this study. Further studies on selective culturing of dominant bacteria, development of probiotic starter cultures and standardisation of processing methods may lead to industrialisation of ethnic food products.

## Materials and Methods

### Sampling

Fifty-four samples of naturally fermented milk products (*chhurpi*, *churkam dahi* and *gheu*/*mar*) were collected from high altitude mountains (1650–2587 meter) in Arunachal Pradesh (n = 35) and hills and mountains (381–4878 meter) in Sikkim (n = 19) of India (Table 2). The products were aseptically collected from the traditional production centres, transported in an ice-box and stored in the laboratory at −20 °C.

**Table 2:** Sample details of the NFM products of India.

Sample	Sample Code	Animal	State	Region/District	Location	Altitude (meter)	pH
*Chhurpi*	Ch1Cc	Cow	Arunachal Pradesh	Tawang	Cheghar	1705	5.32 ± 0.01
	Ch1Sc			Tawang	Samchin	1650	5.32 ± 0.02
	Ch1Tc			Tawang	Tawang	2587	5.33 ± 0.02
	Ch2Bc			West Kameng	Dirang	2095	5.35 ± 0.01
	Ch2Tc			Tawang	Tawang	2587	5.32 ± 0.01
	Ch6Bc			West Kameng	Bomdila	2339	5.33 ± 0.01
	SCCD		Sikkim	West Sikkim	Dentam	1500	6.05 ± 0.01
	SCCLG			South Sikkim	Lingee	1370	6.03 ± 0.02
	SCCNT			East Sikkim	Nimtar	619	5.89 ± 0.01
	SCCPK			East Sikkim	Pakyong	1120	6.03 ± 0.01
	SCCS			East Sikkim	Singtam	381	5.89 ± 0.01
	SCCTH			West Sikkim	Thingling	1780	5.89 ± 0.01
	SC1CYG			South Sikkim	Yangang	1370	6.11 ± 0.02
	Ch1By	Yak	Arunachal Pradesh	West Kameng	Dirang	2061	5.42 ± 0.02
	Ch3Ty			Tawang	Tawang	2587	5.35 ± 0.01
	Ch4Ty			Tawang	Tawang	2587	5.41 ± 0.01
	Ch5By			West Kameng	Bomdila	2340	5.42 ± 0.01
	SC1YYS		Sikkim	North Sikkim	Yumesamdong	4878	5.87 ± 0.03
	SC2YYS			North Sikkim	Yumesamdong	4878	5.88 ± 0.02
	SC3YYS			North Sikkim	Yumesamdong	4878	5.89 ± 0.01
	SC4YYS			North Sikkim	Yumesamdong	4878	5.90 ± 0.01
*Churkam*	Ck1Bc	Cow	Arunachal Pradesh	West Kameng	Bomdila	2339	5.71 ± 0.01
	Ck1Kc			Tawang	Kudung	1695	5.71 ± 0.01
	Ck1Sc			Tawang	Samchin	1650	5.72 ± 0.01
	Ck1Tc			Tawang	Tawang	2587	5.71 ± 0.01
	Ck2Bc			West Kameng	Bomdila	2339	5.72 ± 0.01
	Ck2Kc			Tawang	Kudung	1695	5.73 ± 0.01
	Ck2Sc			Tawang	Samchin	1650	5.72 ± 0.01
	Ck3Kc			Tawang	Kudung	1695	5.72 ± 0.01
	Ck3Sc			Tawang	Samchin	1650	5.72 ± 0.01
	Ck4Bc			West Kameng	Dirang	2095	5.74 ± 0.01
	Ck4Sc			Tawang	Samchin	1650	5.71 ± 0.01
	DCCLA		Sikkim	North Sikkim	Lachung	2700	6.34 ± 0.03
	Ck1Ty	Yak	Arunachal Pradesh	Tawang	Tawang	2587	5.82 ± 0.01
	Ck5By			West Kameng	Bomdila	2340	5.82 ± 0.01
	Ck6By			West Kameng	Bomdila	2340	5.87 ± 0.02
*Gheu*/*Mar*	Gh1Bc	Cow	Arunachal Pradesh	West Kameng	Dirang	2088	6.53 ± 0.02
	Gh3Kc			Tawang	Kudung	1695	6.52 ± 0.01
	Gh3Sc			Tawang	Samchin	1650	6.52 ± 0.01
	Gh4Cc			Tawang	Cheghar	1705	6.55 ± 0.01
	Gh5Bc			West Kameng	Dirang	2095	6.53 ± 0.01
	Gh5Tc			Tawang	Tawang	2587	6.55 ± 0.02
	Gh7Bc			West Kameng	Bomdila	2339	6.53 ± 0.01
	Gh2By	Yak		West Kameng	Bomdila	2339	6.62 ± 0.01
	Gh2Ty			Tawang	Tawang	2587	6.62 ± 0.01
	Gh4By			West Kameng	Dirang	2102	6.56 ± 0.02
	Gh6Ty			Tawang	Tawang	2587	6.61 ± 0.01
	GH1YYS		Sikkim	North Sikkim	Yumesamdong	4878	6.62 ± 0.01
	GH2YYS			North Sikkim	Yumesamdong	4878	6.63 ± 0.01
	GH3YYS			North Sikkim	Yumesamdong	4878	6.63 ± 0.01
*Dahi*	DHCLA	Cow	Sikkim	North Sikkim	Lachung	2700	4.14 ± 0.02
	DHCT			East Sikkim	Tadong	1649	4.23 ± 0.02
	DHCTH			West Sikkim	Thingling	1780	4.12 ± 0.02
	DHYYS	Yak		North Sikkim	Yumesamdong	4878	4.33 ± 0.02

### Metagenomic DNA extraction

Metagenomic DNA was extracted by two different methods based on the nature of the samples i.e., lipid-rich sample (*gheu/mar)* and casein-based samples (*dahi, chhurpi* and *churkam*). For the *gheu*/*mar* (lipid-rich) samples, extraction of DNA was performed as per method I as described in^48^ with some modifications. This method was chosen on the basis of the product being rich in its fatty content. The usage of a combination of petroleum ether:hexane (1:1) serves the purpose of dissolving the fat content resolving the product into two phases after rigorous vortexing. Briefly, 2 mL of the sample melted in low temperature was homogenized with 2 ml citrate buffer (2%). To this, 4 ml of petroleum ether: hexane (1:1) was added followed by vortexing and 10 min incubation at room temperature. 2 mL of the lower part of the homogenate was transferred to a sterile 2 ml screw-cap tube containing 0.5 g of zirconia/silica beads (0.1 mm) and 4 glass beads (2 mm). The tubes were centrifuged and the pellet resuspended in 150 µl proteinase-K buffer [50 mM Tris-Cl, 10 mM EDTA (pH 8), 0.5% (w/v) SDS]. After overnight incubation at 65 °C with 25 µl proteinase K (25 mg/ml), it was treated with 150 µl of 2X breaking buffer [4% Triton X-100 (v/v), 2% (w/v) SDS, 200 mM NaCl, 20 mM Tris (pH 8), 2 mM EDTA (pH 8)]. After addition of phenol (pH 8.0), the samples were treated in a bead beater three times (30 sec beating, 10 sec in ice) and further purified with chloroform: isoamyl alcohol mixture (24:1). Lastly, DNA was precipitated with ethanol and the pellet is dissolved in 50 µl of TE buffer (10 mM Tris, 1 mM EDTA).

For the casein-based samples (*dahi*, *chhurpi* and *churkam*), metagenomic DNA was extracted using the method of Keisam *et al*.^41^. This method was shown to recover maximum DNA yield from fermented milks^41^, hence it was also applied in this study. Briefly, 10 g or 10 ml of the samples were mixed with 90 mL 2% sodium citrate buffer and homogenized in a stomacher at 200 rpm for 2 min. *Churkam* (hard-cheese) samples were first grinded into powder before the homogenization. 1.5 mL of the homogenate was transferred to a sterile centrifuge tube and centrifuge for 10 min at 18000 × g. To the pellet, 400 µl TES buffer [50 mM Tris, 1 mM EDTA, 8.7% sucrose] 50 KU lysozyme, 25 U mutanolysin and 20 U lyticase were added and incubated at 37 °C for 1 h. After incubation, proteinase-K (25 mg/mL) was added to the mixture and further incubated at 65 °C for 1 h, followed by addition of GES reagent (5 M guanidine thiocyanate, 100 mM EDTA, and 0.5% sarkosyl). The sample was treated with 7.5 M ammonium acetate followed by purification with choloroform: isoamyl alcohol (24:1). Finally, DNA was precipitated with ethanol and the pellet dissolved in 50 µl of TE buffer (10 mM Tris, 1 mM EDTA). In all cases, absence of contaminating DNA in the laboratory prepared reagents was confirmed by extracting DNA from sterile water and observing negative PCR amplification with universal bacterial primers. The quality (A_260/280_) and quantity of the extracted DNA was checked using a spectrophotometer (NanoDrop ND-1000, USA). DNA was stored at −20 °C until required.

### Barcoded Illumina MiSeq Sequencing

For in-depth bacterial community analysis, barcoded Illumina MiSeq amplicon sequencing targeting the V4-V5 region of the 16 S rRNA gene was conducted as described earlier^49^. The forward primer F563–577 (5′-AYTGGGYDTAAAGNG-3′) and barcoded reverse primers R924–907 (5′-CCGTCAATTCMTTTRAGT-3′) with an 8 bp barcode in its 5′-end was used for sample multiplexing^42^. Each PCR reaction was performed in a total volume of 25 µl with a template-free reaction that acts as a control. The following PCR conditions were used for amplification- initial denaturation (98 °C for 5 min); denaturation (98 °C for 15 sec), annealing (55 °C for 30 sec) and elongation (72 °C for 30 sec). The PCR reaction was run for 28 cycles with a final extension process of 72 °C for 5 min. The 430 bp sized products were separated in a 1.5% agarose gel (w/v) and the target bands were carefully excised from the gel with a sterile scalpel blade and then purified using QIAquick gel extraction kit (Qiagen, New Delhi, India) as per the manufacturer’s instructions. The purified DNA was quantified with Qubit dsDNA BR Assay Kit (Invitrogen) in a Qubit 2.0 fluorometer (Invitrogen, Carlsbad, CA) and the individual were samples pooled in equimolar proportions. The final DNA pool was sent to the NGS facility in Xcelris Genomics (Ahmedabad, India) for paired-end MiSeq sequencing (2 × 300 bp). The raw sequence reads obtained was analysed using the default settings in MG-RAST^43^ and an open-source bioinformatics pipeline QIIME v1.8.0^44^. A total of 7,614,683 post-quality filtered sequences originating from 54 samples belonging to 4 food types of NFM samples were uploaded to MG-RAST server with the MG-RAST ID number 4732361 to 4732414. The reads were subjected to secondary quality filtering to remove non-rRNA sequences before clustering into operational taxonomic units (OTUs) and subsequent generation of OTU tables at four different taxonomic levels (phylum, family, genus and species) using the SILVA SSU database in MG-RAST. Eukaryota-specific and unassigned OTUs were removed before performing further analysis.

### Statistical Analysis

Normalisation of the OTUs relative abundance data was performed by log transformation log_10_ (x_i_ + 1). To understand the variation in the microbial community structure of different food types, PCA was plotted using Canoco software v4.52 (Wageningen University, The Netherlands). Significant difference in the bacterial community structure amongst the four food type was evaluated by ANOSIM with 10,000 permutations using Bray-Curtis similarity index in PAST v2.17. Any significant difference in the abundance of individual taxa at four different taxonomic levels between the four food types was tested by p-value calculation using Student’s twov-tailed paired t-test and ANOVA. p-value < 0.05 was considered statistically significant and the differences in taxon abundance were represented as boxplots using BoxPlotR^45,46^. Species level-OTUs table was rarefied at a depth of 50 to 6482 sequences using the multiple_rarefactions.py script in QIIME for generation of alpha diversities rarefaction curves. Rarefaction plots were generated for Chao1 richness, diversity indices (Fisher alpha, Shannon), Shannon’s equitability and Good’s coverage using the make_rarefaction_plots.py script^44^. Significant differences in the alpha indices amongst the food types were calculated using the script compare_alpha_diversity.py in QIIME.

### Data availability

Sequence data associated with this present work have been uploaded to MG-RAST server with the MG-RAST ID number 4732361 to 4732414.
